# The effects of strawberry cropping practices on the strawberry tortricid (Lepidoptera: Tortricidae), its natural enemies, and the presence of nematodes

**DOI:** 10.1093/jis/14.1.122

**Published:** 2014-09-01

**Authors:** Lene Sigsgaard, Cyril Naulin, Solveig Haukeland, Kristian Kristensen, Annie Enkegaard, Nauja Lisa Jensen, Jørgen Eilenberg

**Affiliations:** 1 University of Copenhagen, Department of Plant and Environmental Sciences, Section of Organismal Biology, Thorvaldsensvej 40, DK-1871 Frederiksberg C, Denmark; 2 Bioforsk, Norwegian Institute for Agricultural and Environmental Research, As, Norway; 3 Aarhus University, Faculty of Science and Technology, Department of Agroecology, Research Centre Foulum, Blichers Alle 20, Postboks 50, DK-8830 Tjele, Denmark; 4 Aarhus University, Faculty of Science and Technology, Department of Agroecology, Research Centre Flakkebjerg, DK-4200 Slagelse, Denmark; 5 HortiAdvice Scandinavia A/S, Hvidkarvej 29, DK-5250 Odense SV, Denmark

**Keywords:** Acleris comariana, Copidosoma aretas, parasitoid, organic farming

## Abstract

Cropping practice can affect pests and natural enemies. A three-year study of the strawberry tortricid, *Acleris comariana* (Lienig and Zeller) (Lepidoptera: Tortricidae), its parasitoid *Copidosoma aretas* Walker (Hymenoptera: Encyrtidae), and its entomopathogenic fungi was conducted in seven pairs of organic and conventional farms to test the hypothesis that farming practice (organic versus conventional) will affect the level of pest infestation and will affect the natural enemies. In addition, the number of years with strawberries on the farm, field age, and other factors that may affect pests and their natural enemies were considered. Farms were characterized by their cropping practices, cropping history, and other parameters. Field-collected larvae were laboratory reared to assess mortality from parasitoids and entomopathogenic fungi. In 2010, a survey of nematodes was made to assess the response of an unrelated taxonomic group to cropping practice. 2,743 larvae were collected. Of those, 2,584 were identified as *A. comariana.* 579 *A. comariana* were parasitized by *C. aretas* and 64 *A. comariana* were parasitized by other parasitoid species. Finally, 28% of the larvae and pupae of *A. comariana* died from unknown causes. Only two of the field-collected *A. comariana* larvae were infected by entomopathogenic fungi; one was infected by *Isaria* sp. and the other by *Beauvaria* sp. The density of *A. comariana* was on average four times lower in organic farms, which was significantly lower than in conventional farms. *A. comariana* was more dominant on conventional farms than on organic farms. The effect of crop age (One, two, or three years) on *A. comariana* infestation was significant, with higher infestations in older fields. Crop age had no effect on *A. comariana* infestation in a comparison of first- and second-year fields in 2010. Cropping practice did not lead to significant differences in the level of total parasitism or in *C. aretas* parasitism; however, *C. aretas* contributed to a higher proportion of the parasitized larvae on conventional farms than on organic farms. Mortality from unknown causes of *A. comariana* was higher in organic farms than conventional farms, and unknown mortality was two to seven times higher in second-generation *A. comariana* than in first generation. Entomopathogenic nematodes were found on three organic farms and one conventional farm. Plant parasitic nematodes were found in more samples from conventional farms than from organic farms. The low density of *A. comariana* in organic farms exposes the specialist *C. aretas* to a higher risk of local extinction. In organic farms, where the density of *A. comariana* is low, other parasitoids may play an important role in controlling *A. comariana* by supplementing *C. aretas* . Other tortricid species may serve as alternative hosts for these other parasitoids, contributing to conserving them in the habitat. The higher unknown mortality of larvae from organic fields may be the result of non-consumptive parasitoid or predator effects. This study reports an example of the effects of cropping practice on an insect pest, with similar effects on nematodes. An understanding of the responsible factors could be used to develop more sustainable cropping systems.

## Introduction

Cropping practice can affect pests and natural enemies with effects on crop health and yield. As a result of reduced use of agrochemicals and more semi-natural vegetation, defined as a habitat within or outside a crop containing a community of non-crop species (J. Holland, Game and Wildlife Conservation Trust, UK, personal communication), on organic farms, organic cropping practice is believed to lead to increased species diversity ( Bengtsson et al. 2005 ) and to increased natural enemy pest control ( Crowder et al. 2010 ).

A three-year study at 14 farms in Denmark was conducted to assess the effects of cropping practice on the strawberry tortricid, *Acleris comariana* (Lienig and Zeller) (Lepidoptera: Tortricidae), and its natural enemies with a focus on the major parasitoid *Copidosoma aretas* Walker (Hymenoptera: Encyrtidae) and entomopathogenic fungi that infect *A. comariana* larvae. We also recorded the presence of plant‒parasitic and entomopathogenic nematodes in soils of the same 14 farms to assess the response of an unrelated taxonomic group of organisms to cropping practice.

A complex of six species of tortricids (Lepidoptera: Tortricidea) can attack strawberry crops. These include the strawberry tortricid *Acleris comariana* , which is the most common species, *Celypha lacunana* (Denis and Schiff), *Clepsis spectrana* (Treits), *Cacoecimorpha pronubana* (Hübn.), *Cnephasia asseclana* (Denis and Schiffermüller), and *Lozotaenia forsterana* ( Bovien and Thomsen 1950 , Stenseth 1982 , Alford 1984 , Cross et al. 2001 ). Although *A. comariana* is one of the main arthropod pests of strawberry, additional major pests include the strawberry weevil *Anthonomus rubi* Herbst, spider mites and tarsonemid mites ( Lindhard et al. 2003 ). Tortricid larvae feed on the leaves and flowers of strawberries. Using pesticides to control moth larvae is harmful to many natural enemies and may contribute to the propagation of other pests, such as mites; therefore, there is a need to look at alternative methods for moth control ( Sigsgaard and Petersen 2008 ). An understanding of the effects of cultivation practice on *A. comariana* numbers and how mortality factors can contribute to the control of *A. comariana* is fundamental to developing conservation biological control strategies.

The main parasitoid of *A. comariana* is the egg‒larval parasitoid *Copidosoma aretas* ( Sigsgaard et al. 2013 ). This parasitoid is the dominant parasitoid of *A. comariana* in the United Kingdom ( Turner 1968 ; Alford 1975 , 1976), yet the effect of cropping practice on this parasitoid is not known. Insect pathogenic fungi have not been recorded for *A. comariana* , but they are known from other Lepidopterans (Guzmán-Franco et al. 2008). Insect pathogenic fungi can be an important mortality factor and subsequently are of relevance for conservation biological control.

Entomopathogenic nematodes occur naturally in the soil (as infective non-feeding juvenile stages) and parasitize the soil inhabiting stages of their insect hosts. Entomopathogenic nematodes are common in cultivated and uncultivated land, and many species occur more frequently in sandy soils ( Hominick 2002 , Griffin et al. 2005 , Haukeland et al. 2006 ). Studies on the natural populations of entomopathogenic nematodes have been concerned mostly with their prevalence in certain habitats or altitudes and the discovery of new species. Few studies, if any, have compared the presence of entomopathogenic nematodes in different cropping systems.

In this study, we aimed to analyse the effect of organic or conventional cropping practices on *A. comariana* and its natural enemies and how these cropping practices affect the interaction between *A. comariana,* its parasitoids, and entomopathogenic fungi. Nematodes were sampled in the same fields to test whether cropping systems affect a taxonomic group unrelated to insects and fungi in a similar way.

## Materials and Methods

### Farms and fields

The project was designed to have six pairs of farms, which matched as closely as possible in terms of geography, area, and number of years with strawberries. Because there are few organic strawberry farms, the organic farms were selected before the conventional farms. Two more farms were subsequently included in the study: in 2009 a large conventional strawberry production (conventional farm 7), and in 2010 an organic farm (organic farm 7). The inclusion of organic farm 7 overlapped in time with farm 3, which it later replaced when organic farm 3 phased out strawberry production in 2010. These two latter farms, conventional 7 and organic 7, constituted the last pair of farms. The farms were selected to represent a range of sizes and cropping histories (e.g., either a long history or on new sites) (see Table 1 ).

**Table 1.**
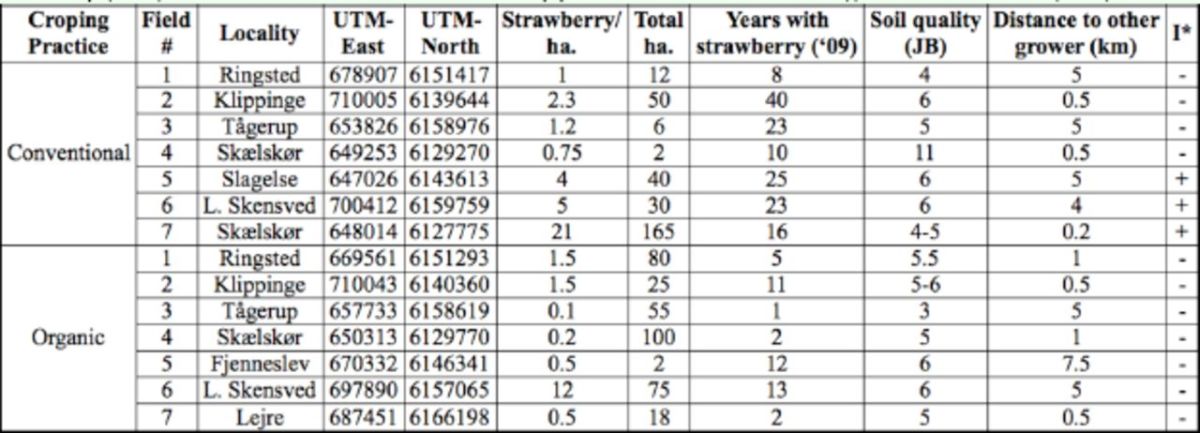
Farms sampled. Cropping practice, number, locality, geographical UTM coordinates (Zealand includes zones 32 and 33, we used the modified map where all of Zealand is in zone 32), area with strawberry, total area belonging to farm, years with strawberry (2009), distance to other farms with strawberry production, use of insecticide (I), stems/m row ± SE (2011).

One field in each farm was studied. The fields selected in 2009 were one-year strawberry, indicating that they had been planted the previous year, and this was the first year of harvest. In 2010, samplings were continued in the same fields, which were then two-year strawberry. The exception was that in farm 7, the field in 2009 was a three-year crop, but it was replaced by a two-year crop in 2010. An assessment in 2010 of tortricid numbers in new one-year crops compared with the two-year crops was made to provide a separate data set to analyse the effect of crop age on tortricid infestation. The new one-year crops replaced the old fields for samplings of the summer generation in 2010 in those cases where the two-year crop had been removed. In spring 2011, conventional farms 2, 3, and 7 and organic farm 7 had three-year-old fields and the remaining fields were two-years old.

### Field assessments 2009‒2011

Farms were characterized according to cropping practice, cropping history, area dedicated to strawberry cultivation, and proximity to other strawberry farms. Tortricid infestation was recorded from April 2009 to June 2011, covering five generations of *A. comariana* . In 2009, spring larvae were sampled only once in early May in one-year fields when larvae were about third to fifth instar. One field was sampled in each farm ( Table 1 ). When possible, fields with the widely used early strawberry, *Fragaria × ananassa* Duch. variety ‘Honeoye,’ were selected to reduce variation. Thirty random samples were made during a diagonal transect walk of the field. In the following samplings, two collections were made per generation, when the larvae were second to third instar and again when they reached fourth to fifth instar. Subsequent samplings included least 30 samples, and this was increased up to a maximum of 60 samples when larval density was low. If necessary, more samples were obtained by using an additional diagonal transect walk, which created a sampling path in the form of an X. Infestation was recorded by registering the number of larvae per 1 m of row, equivalent to 3–3.5 Honeoye plants.

The summer larvae of 2009 were sampled twice, with a 10-day interval, in late July and early August. Sampling continued in spring and summer of 2010 and spring of 2011. In 2010, a one-year field was also sampled at each farm to allow a direct comparison of the effect of field age on infestation.

### Parasitism of *A. comariana*

To assess mortality caused by parasitoids and entomopathogenic fungi, larvae were reared individually in 35 mL containers at 20 ± 1°C. The bottom of each container was covered with 1% water agar. A strawberry leaf was inserted in the agar for the larva to feed on. Larvae were transferred to a new container with a fresh leaf twice a week. The larvae were examined every second day, and any mortality was recorded.

Emerged parasitoids were preserved for later determination. *Copidosoma aretas* was identified to species (Guerreri and Noyes 2005); other parasitoids were determined to the family level only. Dead larvae without signs of parasitism were incubated to screen for insect pathogenic fungi. Field-collected larvae that emerged as adults were likewise identified to species. Finally, pupae that did not emerge were dissected.

### Prevalence of insect pathogenic fungi.

Dead larvae without signs of parasitism were placed in humid chambers to enhance fungal growth. Spore preparations were made on a microscope slide for identification ( Lacey 1997 ). Normally infestation is clearly visible after two weeks, but to ensure that no pathogens were missed, all samples were retained for three weeks; others, for which there was some doubt, were retained for up to five weeks.

### Influence of cropping practice on nematodes

In mid-September 2010, nematodes were sampled in the same fields from which the insect samples were collected, providing seven organic and seven conventional samples. Samples were taken using a soil corer. Each sample comprised 20 pooled cores of 2.5 cm diam taken to a depth of -15 cm. Cores were taken as evenly as possible along a “W” pattern within each field and mixed to give one sample of **-** 1 kg/ field.

To recover entomopathogenic nematodes, a subsample from each soil sample was placed in a 500 mL plastic container and baited with three late larval instars of *Galleria mellonella* L. (Lepidoptera: Pyralidae) ( Kaya and Stock 1997 ). The containers were held at room temperature, and the larvae were examined for mortality and entomopathogenic nematode infection after one and two weeks. Dead larvae were placed in humid Petri dish chambers to allow for nematode development. Nematodes were identified to genus by morphological examination of the infective juvenile stages ( Adams and Nguyen 2002 ). Free-living plant parasitic nematodes were extracted from a 250 mL sub-sample from each soil sample using the elutriation technique ( Seinhorst 1962 ). Plant parasitic nematodes were identified to genus by morphological examination of the adult stages.

### Yield

Strawberry yield was assessed at all farms in 2011 in the same fields as used in the study. In late June, the number of flowers, fruit, and empty stems were counted in eight to 10 randomly selected 1 m rows, equivalent to 3-3.5 plants. The sum of flowers, fruit, and empty stems represented the production potential of the crop. This measure replaced a yield estimate because frost damage affected fruit formation of several fields in 2011.

### Data analysis

**Prevalence of** ***A comariana*** **in the field.** The number of observed tortricids was summarized for each field. Data (sum of larvae per field per sampling date) were then analysed in a generalised linear mixed model, where the number of larvae, based on analyses for best fit, was found to be Poisson distributed with an over dispersion within each year and pair. The mean depended on cropping practice, year, crop age, and generation, as well as interaction between these (as systematic effects). In addition, we attempted to include characteristics for the individual farms/fields as explanatory variables, with the possibility that the effect depended on the cropping practice. The farms were ordered in pairs, and we attempted to include one organic and one conventional farm in each pair. These pairs and year, field, and generation within field were included as a random effects. The over-dispersion parameter and random effects were included to take into account variations not caused by random sampling, for example, variation caused by soil and environmental effects within and between fields. Finally, the over-dispersion effect was estimated and taken into account. To accommodate the slightly varying numbers of samples per field, the logarithm to the number of samples was included as an offset-variable. The offset-variable also included the correction needed to account for sampled tortricids that were not *A comariana.*

**Mortality factors.** The proportion of *A. comariana* larvae parasitized by *C. aretas* and by other parasitoids as well as the proportion of deaths and adult emergence were analysed using a generalized linear mixed model assuming proportion parasitized, proportion dead, and proportion of adult emergence to follow a binominal distribution with an over-dispersion within each year and pair. In the model, the logit of the binomial probability depended on the fixed effects of cultivation method, generation, year, the two-way interactions among these, and the random effects of pair and pair within year. Finally the over-dispersion effect was estimated and taken into account. The over-dispersion parameter and random effects were included for the same reason as mentioned in section above; however, nonsignificant interaction effects of fixed effects were removed from the final model.

**Nematodes.** To analyze the effect of cropping practice on soil parasitic nematodes, soil samples were ranked according to presence of plant parasitic nematodes: 0 indicated no plant parasitic nematodes were present; 1 indicated the presence of either *Pratylenchus* or *Longidorus;* and 2 meant that the plant parasitic nematode genera co-occurred. For the entomopathogenic nematodes belonging to the genus *Steinernema,* samples were by the nature of the baiting method either 0 in cases where the beneficial was not found or 1 when it was present. Data for both groups of nematodes were analyzed using the Wilcoxon signed rank test. Data for plant parasitic nematodes *Pratylenchus* and *Longidorus* were also analysed in a generalised linear mixed model; the number of nematodes, based on analyses for best fit, was found to be Poisson distributed with a mean that depended on cropping practice. We attempted to include additional characteristics for the individual fields as explanatory variables, with the possibility that the effect depended on the cropping practice. The farms were ordered in pairs with one organic and one conventional farm in each pair. These pairs were included as a random effect.

**Yield.** Yield was analysed using a linear mixed model, with cultivation method as fixed effects and pairs together with farms as a random factor.

## Results

### Effect of cropping practice and other management practices on tortricid infestation of strawberry

**Figure 1.**
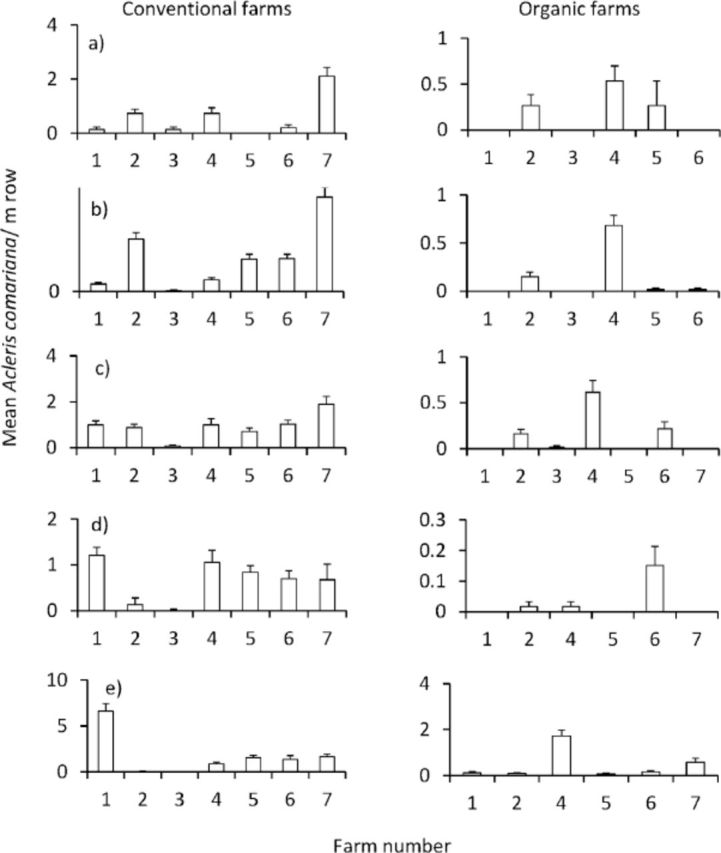
Field densities of *Acleris comariana* in organic and conventional strawberry fields from 2009-11. Columns to the left are the conventional fields, columns to the right the organic fields, (a) spring 2009, (b) summer 2009, (c) spring 2010, (d) summer 2010, (e) spring 201 1. High quality figures are available online.

In this study, 2,743 tortricid larvae were collected. Of these 1,198 adult *A. comariana* emerged. 159 other tortricids were found, some of which completed development to adults: these were *Celypha lacunana* Denis and Schiffermüller *(n =* 40 adults, 22 from conventional fields), *Cnephasia asseclana* Denis and Schiffermüller *(n =* 49 adults, 20 from conventional fields), and *Clepsis spectrana* Treitschke *(n =* 2, both from conventional fields). The larvae of these species are normally darker than *A. comariana* larvae and can be distinguished from *A. comariana* in the laboratory. The larvae of the three other tortricid species feed on a variety of herbaceous plants. The densities of *A. comariana* per meter row, equivalent to 3–3.5 plants for the study period, are presented in Fig. 1 , which is corrected for the presence of other tortricids.

The densities of *A. comariana* , corrected for the presence of other tortricids, depended strongly on cropping practice (organic or conventional) ( Fig. 1 ), with higher densities under conventional practice (F1,24.6 = 14.75 , *P* = 0.001). No significant main effect was found for the year of study or generation (spring, summer). There was a significant effect of crop age (one, two, or three year) (F2,45.8 = 3.43, *P* = 0.041), with a higher infestation in older fields. Interaction effects were nonsignificant. One explanatory variable was found to be significant and was included in the analysis: Universal Transverse Mercator (UTM) North (F1,8.7 = 11.1, *P* = 0.008), the log( *x* ) estimate being (‒0.00007 ± 0.00022), indicating that the numbers of *A. comariana* decreased by 0.07 times per km north. One other explanatory variable, the number of years a farm had produced strawberries, was near significant (F1,14.7 = 3.48, *P* = 0.082).

Insecticide use was not a significant explanatory variable for densities of *A. comariana* . Conventional farms 5, 6, and 7 used insecticides in strawberries. Pyrethroid was applied in spring or early summer to control *A. comariana* or *A. rubi* , or both. The remaining conventional farms used only herbicides and fungicides, except for conventional farm 3, which did not use herbicides. All of the conventional farms used fertilizer.

*A. comariana* was the dominant tortricid in organic and conventional farms, but less so in organic farms. In organic farms, *A. comariana* made up 85.2% of the individuals, and in conventional fields it made up 96.9% of the individuals (F1,48 = 31.7, *P* < 0.0001). The interaction effect of the time of sampling (following the two generations of *A. comariana* ) and the year on proportion of other species was also significant (F1,48 = 4.3, *P* = 0.04). Thus, in 2009, other tortricid species made up 2.7% of the individuals collected during sampling for the first generation, whereas other tortricids made up only 0.4% of the individuals sampled for the second generation, equivalent to seven times less. For 2010, the corresponding percentages were 6.2% and 3.3%, equivalent to half as many other tortricids found while sampling for the second generation. In 2011, only the first generation was sampled, and 11.0% of the individuals in this sampling belonged to other tortricid species.

### Effect of cropage

In 2010, we sampled new first-year strawberry fields at the same time as the second-year fields, once for both generations (the second sampling), to test whether crop age influenced infestation. An analysis of the density of *A. comariana* as an effect of cropping practice, generation (first or second), crop age, and all interaction effects showed that the average number of *A. comariana* per m row found in the first-year fields (conventional 0.63 ± 0.14, organic 0.14 ± 0.05) was not significantly different from that in the second-year fields (conventional 0.81 ± 0.14, organic 0.11 ± 0.05) (F2,36 = 0.55, *P* = 0.584), whereas there was a highly significant effect of cropping practice (F1,21.1 = 11.13, *P* = 0.003), with a higher density of *A. comariana* in conventional fields (lsmeans conventional: 11.9 ± 1.9, organic 2.8 ± 1.9) and a significant effect of generation (lsmeans first generation 9.1 ± 1.7, second generation 5.7 ± 1.7) (F _1,23.5_ = 4.71, *P* = 0.040). There were no significant interaction effects.

### Natural parasitism of *A. comarian*

**Figure 2.**
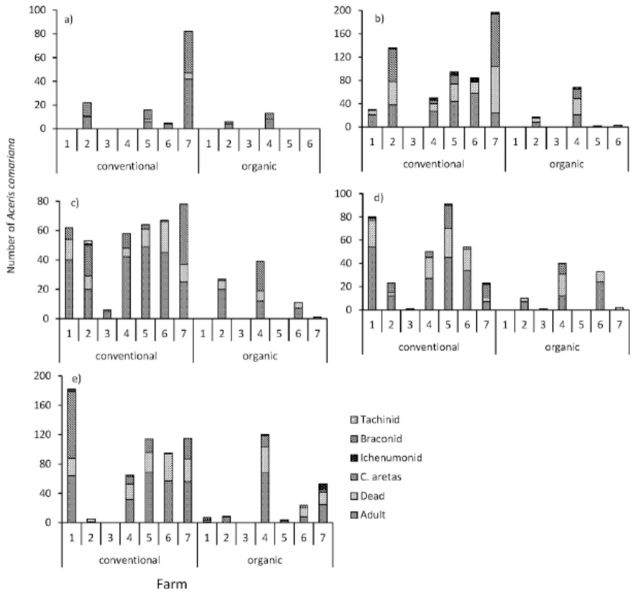
Mortality factors of *A. comariana* for organic fields number 1 to 7 and conventional fields number 1 to 7 *.* The height of stacked columns shows the total number of larvae collected from each field. From below they are: the number emerging as adult *A. comariana* (50% grey), number dead (hatched lower left to upper right), parasitized by *C. aretas* (hatched, bold line, upper left to lower right), Ichneumonidae (80% black), Braconidae (chequered), Tachinidae (zigzag lines). (a) 2009 first generation, which was sampled only once; all subsequent generations were sampled twice; (b) 2009, second generation; (c) 2010 first generation; (d) 2010 second generation; (e) 2011, first generation. High quality figures are available online.

The dominant parasitoid was *C. aretas* . Of the *A. comariana* larvae collected, 579 were parasitized by *C. aretas* . Other parasitoids were recovered from 64 *A. comariana* larvae or pupae *.* They were primarily Hymenopterans (Ichneumonidae [31] and Braconidae [15]) but also included a few Dipterans (Tachinidae [6]). Dissection of un-emerged pupae showed that 12 were parasitized by hymenopterans, which had died before emergence. Finally, 664 larvae and 122 pupae died from unknown factors, i.e., neither parasitism nor fungal pathogens could be detected. The number of larval deaths from unknown factors was equivalent to an unknown mortality of 28% ( Fig. 2 ).

**Figure 3.**
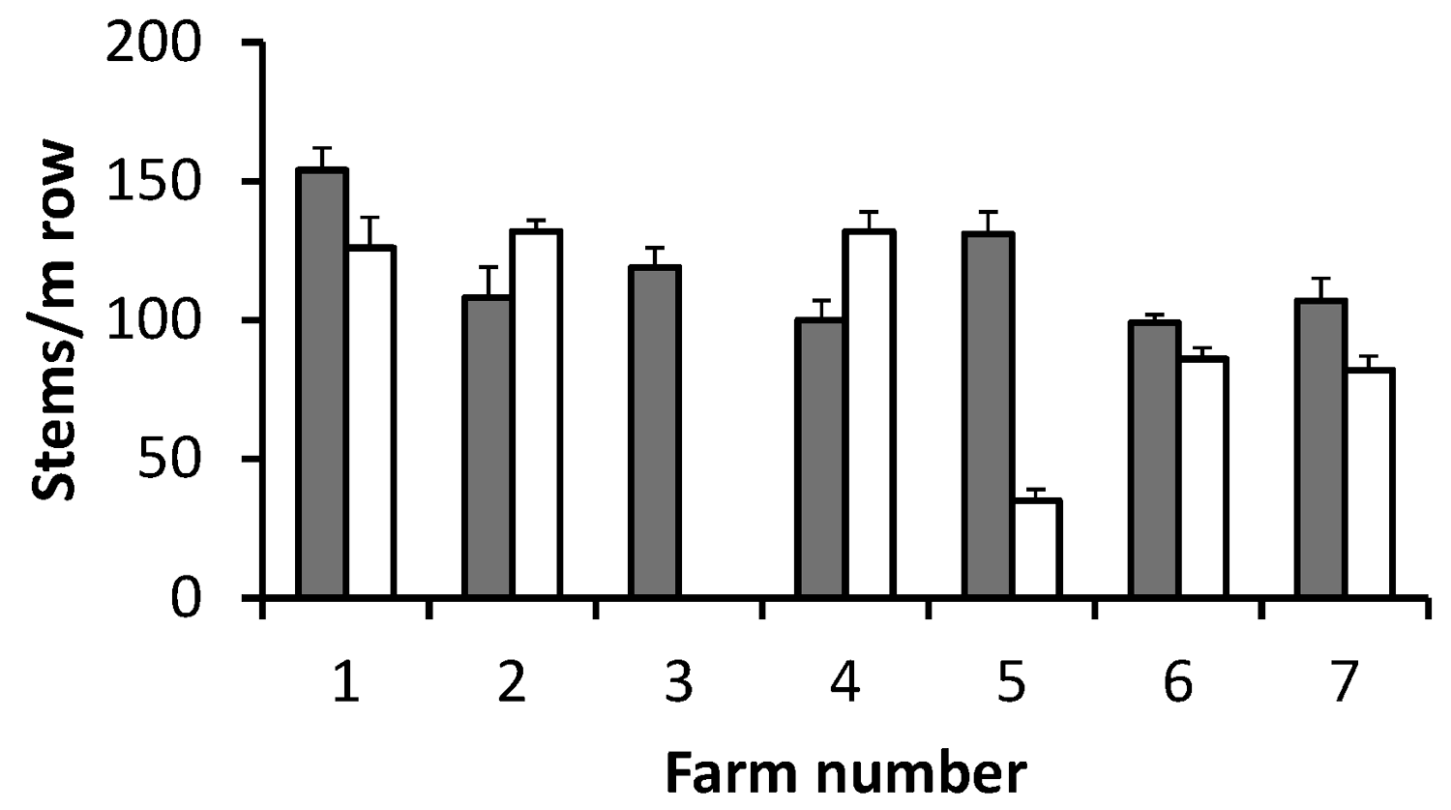
Production potential of strawberry in seven conventional and seven organic farms in 201 1, represented by averaged total number of flowers, fruit and empty stems, i.e. stems per 1 m row, counted in 8-10 randomly selected 1 m rows, equivalent to 3-3.5 plants in late June. This measure replaced a yield estimate because frost damage affected fruit formation of several fields. High quality figures are available online.

An analysis of the proportion of *A comariana* adults emerging from collected larvae as a function of cropping practice (organic or conventional), generation (first or second), and year (2009, 2010, 2011) (Proc Glimmix, SAS Institute, www.sas.com ) showed a significant main effect of generation, reflecting that the successful emergence in first generations were higher (51.7%) than in second generations (42.8%) (F _1,32.3_ = 5.83, *P* = 0.022), and no interaction effects were significant.

The proportion of larvae that died of unknown causes was analysed as a function of cropping practice (organic or conventional), generation (first or second), and year (2009, 2010, 2011) (Proc Glimmix). There was a significant main effect of cropping practice on unknown mortality, with a higher mortality in the second generation. In conventional farms, it was 20.9% in the first generation and 30.7% in the second generation; in organic farms, it was 26.2% in the first generation and 39.7% in the second generation (F _1,44_ = 5.08, *P* = 0.029). There was also a significant interaction effect of year and generation on mortality. In 2009, it was 6.2% in the first generation and 33.3% in the second generation. In 2010, it was 19.7% in the first generation, and 30.4% in the second. In 2011, when only the first generation was sampled, mortality was 26.4% (F _1,44_ = 17.53, *P* < 0.001).

A near-significant higher proportion of larvae were parasitized by *C. aretas* in conventional farms (24.9%) than in organic farms (15.8%) (F _1,46_ = 3.9, *P* = 0.054). There was also a near-significant difference among the three year crops —30% individuals were parasitized by *C. aretas* in 2009, 18% in 2010, and 21% in 2011 (F _1,46_ = 3.9, *P* = 0.054).

There was no difference in total parasitism of *A. comariana* between conventional farms (26.8%) and organic farms (21.0%) (F _1,46_ = 0.04, *P* = 0.839). The main effects of year and generation were also not significant. However, the proportion of total parasitism of *A. comariana* by *C. aretas* was higher in conventional farms (93.1%) than in organic farms (74.3%) (F _1,34_ = 15.0, *P* < 0.001).

### Natural occurrence of insect pathogenic fungi (prevalence)

Only two of the incubated larvae were naturally infected with insect pathogenic fungi. One larva, collected in 2011 from conventional farm 6, was infected by a species of *Beauveria* . The second larva, also collected in 2011 but from conventional farm 7, was infected by a species of *Isaria* . Both isolates are maintained in the fungal culture collection at the Department of Plant and Environmental Sciences at the University of Copenhagen.

**Table 2.**
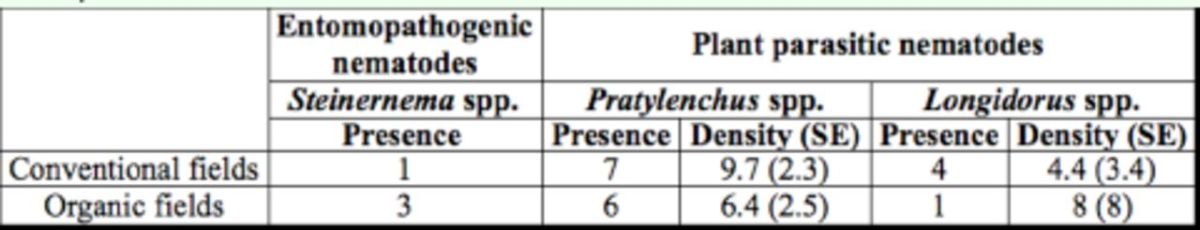
Presence of entomopathogenic nematodes and plant parasitic nematodes (number of samples (fields) with nematodes) and density of plant parasitic nematodes (mean number of nematodes per 250 mL soil) in seven conventionally and seven organically grown strawberry fields.

### Effect of cropping type on nematode population

Entomopathogenic nematodes belonging to the genus *Stenernema* were recovered from one conventionally grown field and from three organically grown fields. Plant parasitic nematodes were also present. The dominant plant parasitic nematode genera were *Pratylenchus* and *Longidorus* . *Pratylenchus* was present in seven conventionally grown fields and six organically grown fields; *Longidorus* was present in four conventionally grown fields and one organically grown field ( Table 2 ). The presence of entomopathogenic nematodes was not significantly different between organic and conventionally grown fields (Wilcoxon S = ‒2.5, P = 0.62). Also the presence of plant parasitic nematodes (ranked) was not significantly higher in conventional fields than in organic (Wilcoxon S = 3, *P* = 0.25). A mixed linear model of total number of plant parasitic nematodes as a function of cropping practice with farm pair as a random variable and with years with strawberry and soil textural class as covariables showed no significant interaction effects, a near-significant effect of soil textural class (F _1,__6.3_ = 5.18, *P* = 0.061), and no significant effect of cropping practice (F _1,8.1_ = 0.92, *P* = 0.366).

### Yield

The yield of the 14 fields in 2011, excluding organic field 3, which was no longer cultivated in 2011, is presented ( Table 1 ) as the sum of flowers, fruit, and empty stems per m row by late June. This number, which represents potential rather than actual yield, was used because frost damage made it impossible to compare harvested yield. Yield was not significantly different in conventional and organic fields (F _1,11_ = 1.2, *P* = 0.30). A model including *A. comariana* density as an explanatory variable was not significant.

## Discussion

Across the three years of the study, there was a highly significant lower infestation of *A. comariana* in organic strawberry fields. Also, there was a significant effect of crop age. No other factors were significant, and there were no interaction effects. A single covariable, UTM-north, was significant, with infestation of *A. comariana* decreasing from south to north by 0.07 times per km. It is normally assumed that problems in strawberry fields build up over time, but multiple years with strawberry production on a farm was only a near-significant covariable and of much less significance than cropping practice. Comparisons between organic and conventional cropping practice at the farm scale are more heterogeneous than studies at plot scale, as found in a metaanalysis ( Bengtsson et al. 2005 ). Such heterogeneity can mask the effect of covariables but does not exclude an influence by them.

Although we found an effect of crop age during the three-year study, when we compared *A. comariana* infestation in first- and second-year fields within individual farms, no difference in infestation level was found. This may be because field distances between old and young strawberry crops within farms were small. In fact, it is still a common practice to plant old and young strawberry crops next to each other. Young and old crops may thus be separated by just a few meters, especially on farms where strawberry is a minor product, whereas distances of up to 100 m between young and old strawberry crops existed on the large farms. Short distances facilitate the migration of insects between old and young crops. If the effective mobility of *A. comariana* is restricted to a few hundred meters, as noted by Turner (1968) and as indicated for tortricids in general ( Jeanneret and Charmillot 1995 ), a farm layout with larger temporal and spatial distances between strawberry crops could, in all likelihood, reduce this tortricid pest.

*A. comariana* was most dominant in conventional farms. Although the actual number of other tortricid species was comparable between organic and conventional farms, the proportion of these other tortricid species in organic strawberry fields was significantly higher. These other tortricid species were generalists with a wide host-plant range and it is likely that they originated in the surrounding landscape or other crops. The higher nutrient levels normally found in conventional fields are predictably correlated with higher densities of generalists ( Staley et al. 2010 ); however, our findings do not agree with this.

Although total parasitism of *A. comariana* was not different between the two cropping practices, the proportion of larvae parasitized by *C. aretas* was almost significantly higher in conventional farms than in organic farms. The percentage of parasitism by *C. aretas* in this study (organic 16%, conventional 25%) is higher than the < 10% reported by Turner (1968) and Vernon (1971) but lower than the 76% found by Alford (1976) .

The proportion of parasitism by *C. aretas* was highest in conventional farms, with a higher contribution of parasitism by other parasitoids in the organic farms. A strong correlation between *A. comariana* density and parasitism by *C. aretas* underlines the potential of this parasitoid to regulate *A. comariana* ( Sigsgaard et al. 2013 ) **;** it can also help to explain the low density of this parasitoid in organic farms. Where the density of *A. comariana* is low, the specialist *C. aretas* is exposed to a higher risk of local extinction. For those systems in which extinction probabilities have been quantified, the parasitoid is more prone to extinction than its host, supporting the theoretical expectation that higher trophic levels are at greater risk of extinction than lower trophic levels ( Cronin and Reeve 2005 ).

Results indicate that at low densities of *A. comariana* , as in organic farms, other parasitoids provide supplementary control to that of the specialist *C. aretas* . Other tortricid species may serve as alternative hosts for these other parasitoids, which in turn may serve to maintain these other parasitoids in the habitat, e.g., the parasitoid *Colpoclypeus florus* (Walker) of leafrollers in apple ( Pfannenstiel et al. 2010 ).

The higher unknown mortality of *A. comariana* larvae collected in organic farms calls for further study. Identical rearing conditions for all collected larvae exclude the possibility that this was an effect of rearing. A possible unknown mortality factor could be non-consumptive predator effects ( McCauley et al. 2011 ) or from parasitoid probing. The higher unknown mortality in the second generation of *A. comariana* , during a period of the summer when there are more natural enemies, could support this hypothesis.

The lower dominance of *A. comariana* and *C. aretas* in organic fields is in accordance with the findings of a meta-study on the effect of organic farming practice on diversity and further demonstrates that there is a higher diversity associated with organic farming practices ( Bengtsson et al. 2005 ). Likewise, in a comparison of parasitoid diversity between 10 organic and 10 conventional farms, Macfadyen et al. (2011) found significantly more parasitoid species on organic farms, with more species in each functional group.

The higher diversity of tortricids and parasitoids in organic fields found in this study may be the result of higher floral diversity than in conventional fields. For *A. comariana* and *C. aretas,* it is known that flowering plants may increase the longevity of both species ( Sigsgaard et al. 2013 ) **,** which in turn may lead to higher densities. The abundance of flowering plant species and species richness within strawberry fields and hedgerows of conventional farms 1, 2, 3, 5, and 7 and organic farms 1, 2, 4, and 7 according to a study by E. J. Ahrenfeldt et al. (University of Copenhagen, Denmark) is comparable. Thus the floral species richness in conventional fields was 12, 12, 7, 5, and 7 plant species, respectively; and in the four studied organic fields, plant species richness was 13, 10, 3, and 10 species, respectively. Richness in conventional hedgerows adjacent to the conventional fields was 6, 13, 7, 5, and 6 plant species, respectively, and in organic hedgerows was 9, 9, 6, and 6 species respectively. Differences in plant or crop diversity at larger scales, such as between fields, cannot be excluded.

Organic cropping practice is thought to lead to increased species diversity (Bengtsson et al. 2005) and to increased natural enemy pest control ( Crowder et al. 2010 ) as a result of more semi-natural vegetation and a reduction in the use of agrochemicals on organic farms. However, only three of the conventional farms in this study used insecticides, and insecticide was not a significant covariable. All of the conventional farms used fertilizers, herbicides (except one), and fungicides. Herbicides and fungicides may either affect arthropods directly or have indirect effects through changes in floral composition. Noxious effects of herbicides on the eggs of parasitoids have been reported for the encyrtid *Trichogramma pretiosum* Riley ( Bueno et al. 2008 ). Although parasitism was not higher on the organic farms, the higher unknown mortality of larvae sampled from these farms points to a higher activity of natural enemies as one explanation for the much lower infestation by *A. comariana* under organic cropping practice.

### Very low prevalence of entomopathogenic fungi on *A. comariana*

The very low natural prevalence of entomopathogenic fungal infestation of *A. comariana* larvae indicates that natural regulation by this group of natural enemies is not likely. This may be attributed to the fact that *A. comariana* larvae are not in contact with soil, although entomopathogenic fungi can also be found on the phylloplane ( Meyling and Eilenberg 2007 ). No previous studies have assessed the prevalence of entomopathogenic fungal infection of *A. comariana;* however, *Entomophthora* was reported from the congener *A. minuta* (Robinson) ( MacLeod and Müller-Kögler 1973 ), and according to Zimmermann and Weizer (1991) , fungi are often found on tortricid larvae and pupae.

### Effect of cropping practice on nematodes

Data may indicate a higher prevalence of entomopathogenic nematodes in organic strawberry fields, whereas plant parasitic nematode levels appear slightly higher in conventional farms, although not significantly so. A higher presence of entomopathogenic nematodes in organically grown fields may be linked to greater insect host availability in such fields, compared with conventionally grown fields, if chemical measures are implemented in the latter. A parallel Norwegian study found a similar response by entomopathogenic nematodes to cropping practice, with a lower infestation by plant parasitic nematodes in organic fields (N. Trandem, Bioforsk, Norwegian Institute of Agricultural and Environmental Research, Ås, Norway, personal communication). Further studies to elucidate which factors favor the apparent higher occurrence of entomopathogenic nematodes in organically grown fields are worth pursuing. Nematode data from the present study and from a study in Norway (N. Trandem, personal communication) suggest that cropping practices may have comparable effects on this unrelated system. Likewise, Gliessmann et al. (1996) , in a comparison between conventional and organic strawberry production during a three-year conversion, found higher levels of beneficial nematodes in organic fields.

Estimated yield was not different between cropping practices, pointing to the possibility of reduced *A. comariana* infestation and reduced pesticide and agrochemical input without a compromise in yield. In contrast to this study, yields found in fields under conversion to organic production, studied by Gliessmann et al. (1996) , were less, possibly because of a conversion decline.

Findings that *A. comariana* numbers are consistently three to four times lower in organic practices than in conventional cropping practices encourages further study into the responsible factors. Understanding such factors may provide information that can aid in the development of more sustainable cropping practices and conservation biological control strategies. The findings also suggest entomopathogenic nematodes may benefit from organic cropping practice with a trend of lower infestation with entomopathogenic nematodes; however, this remains to be studied further *.* Previous studies at the farm scale, where organic and conventional farms were matched according to landscape structure, also show significant differences in terms of diversity and natural enemies, but they also reflect highly heterogeneous results (Bengtsson et al. 2005). For further studies aimed at improving cropping practices to control *A. comariana,* plot experiments may be needed to obtain clearer results, although large plots are needed to study this mobile insect. The low prevalence of fungi probably excludes the use of fungi in conservation biological control. Entomopathogenic fungi may still have potential for inundation or inoculation biological control; however, this still remains to be tested.
